# Hybrid Carbon Nanotubes/Gold Nanoparticles Composites for Trace Nitric Oxide Detection over a Wide Range of Humidity

**DOI:** 10.3390/s22197581

**Published:** 2022-10-06

**Authors:** Ami Hannon, Wayne Seames, Jing Li

**Affiliations:** 1 KBR Wyle Inc. at NASA Ames Research Center, Moffett Field, CA 94035, USA; 2 Department of Chemical Engineering, University of North Dakota, Grand Forks, ND 58201, USA; 3 NASA Ames Research Center, Moffett Field, CA 94035, USA

**Keywords:** chemiresistive sensor, carbon nanotubes, nitric oxide sensor, gold nanoparticles and carbon nanotubes, ppb concentration detection, NOx sensor

## Abstract

Composites of functionalized single walled carbon nanotubes (SWCNTs) and gold nanoparticles (Au NPs) of ≈15 nm diameter were drop-cast on a printed circuit board (PCB) substrate equipped with interdigitated electrodes to make a hybrid thin film. Addition of Au NPs decorated the surface of SWCNTs networked films and acted as catalysts which resulted into an enhanced sensitivity and low ppb concentration detection limit. The compositions of the film were characterized by scanning electron microscope (SEM). SWCNTs clusters were loaded with various amount of Au NPs ranging from 1–10% (by weight) and their effect on Nitric oxide (NO) sensitivity was studied and optimized. Further, the optimized composite films were tested in both air and nitrogen environments and as well as over a wide relative humidity range (0–97%). Sensors were also tested for the selectivity by exposing to various gases such as nitrous oxide, ammonia, carbon monoxide, sulfur dioxide and acetone. Sensitivity to NO was found much higher than the other tested gases. The advantage of this sensor is that it is sensitive to NO at low ppb level (10 ppb) with estimated response time within 10 s and recovery time around 1 min, and has excellent reproducibility from sensor to sensor and works within the wide range of relative humidity (0–97%).

## 1. Introduction

Nitric oxide detection has a wide application from environmental monitoring, industrial process control, combustion studies, oceanographic study to medical diagnoses [1,2,3,4].

While it is possible today to measure trace gases such as NO, Nitrous oxide (N_2_O), and Dimethyl sulfide (DMS) in the atmosphere, the sensors to measure dissolved gases in seawater real time and in situ are limited to Carbon dioxide (CO_2_), Methane (CH_4_), and hydrogen sulfide (H_2_S) [5]. Measurement of climatically relevant trace gases are necessary to quantify ocean sources and sinks, and to understand their impact on global climate change. Several of these climatically relevant gases are known to be produced under low oxygen conditions, such as the oxygen minimum zones in the open ocean, and ‘dead zones’ in the coastal ocean. Global warming is the working hypothesis for the observed expansion of open ocean Oxygen Minimum Zones (OMZs); increased stratification reduces upper ocean ventilation and Aeration [6,7,8]. Expansion of hypoxia in the coastal zone is linked to eutrophication associated with excess nutrients in river runoff, from sources such as chemical fertilizers applied to farms, fields, and lawns [9]. Marine life becomes highly stressed under hypoxic conditions, and dramatic ecological impacts can occur, including massive kills of fish and shellfish and harmful algae blooms. Longer lasting impacts also occur since juvenile fish are more likely to be affected than mature fish, resulting in detrimental follow-on effects such as economic losses. For Texas, with a doubling in population predicted by 2050, the impact of hypoxia on the coastline’s ecosystem and economy is especially concerning [10]. There is a critical need for a deeper understanding of gas cycling in hypoxic zones. The development of an in situ N_2_O sensor is a priority; N_2_O is a known greenhouse gas and can therefore feedback on global warming via increased OMZ expansion [11,12]. Today highly precise measurement of dissolved gases like N_2_O and NO rely on laboratory-based analyses such as mass spectrometry and gas chromatography [13]. The oceanographic community needs a new, small, low-power, real time dissolved gas sensor that can be tuned to different gases of interest to allow high spatial resolution sampling for specific gases of interest. A platform independent sensor can be used on floats, gliders, CTDs, and AUVs for open water and coastal surveys of dissolved gases. In addition, NO has been recognized as an atmospheric pollutant and a potential health hazard. The Occupational Safety and Health Administration (OSHA) have set a permissible exposure limit (PEL) for nitric oxide gas at 25 ppm. Another application is for medical diagnosis, NO concentration variation in breath can signify neuro degenerative diseases or lung related dieses [14,15]. An average, healthy person has an exhaled NO concentration of 6.7–16.2 parts per billion (ppb), while that of an asthma patient has a concentration in the range of 34.7–51.1 ppb [16]. For all these reasons a detector is required which can detect lower concentration of NO selectively and reliably.

Nitric oxide (NO) is a gaseous, free radical, highly unstable and a reactive molecule and detection of such molecules is relatively difficult especially at low concentrations and in high humidity environment [17]. Currently most commonly used techniques to monitor NO gas concentration are electrochemical devices, electron magnetic resonance spectroscopy, chemiluminescence analyzer, transistor-based devices and X-ray photoelectron [18,19,20]. Some of these techniques are designed to detect NO in solutions in vivo and not in situ; other instruments are highly sensitive, accurate and in situ but generally bulky and complicated. There is a great need of development of a new technology-based device that can detect NO selectively in real time with small footprint, hand-held, easy to operate, and consume less power. Chemiresistive sensor technology seems a good fit to satisfy these needs.

There have been a few studies on NO sensing with most of them using metal oxides such as WO_3_, Cr_2_O_3_, In_2_O_3_, ZnO, and SnO_2_ [21,22,23,24,25,26,27]. Metal oxide sensors operate at higher temperatures (350–800 °C) needing a heating element with high-power consumption, and experience poor selectivity. Some researchers also studied conducting polymers such as polyethylene imine, polyaniline, Polyaniline/WO_3_ and reported good sensitivity, however these studies were only performed in either dry or limited humidity environment [4,28]. Recently, single-walled carbon nanotubes (SWCNTs) sensors have been demonstrated to detect NO at room temperature [4,29]. SWCNTs receiving considerable attention because of their outstanding structural, electrical, optical, mechanical, and thermal characteristics [30,31]. However, sensors made of pristine CNTs are often unable to detect certain gases with desirable sensitivity and selectivity. The lack of sensitivity and selectivity can be overcome by functionalizing the nanotubes. CNTs sensors for air pollutants such as NOx and NO_2_ gases are well studied but for NO gas studies are limited. Researchers have used polymer-coated CNT_S_, SWCNTs-COOH, MWCNTs-COOH, (3-aminopropyl) triethoxysilane-SWCNTs, and PEI-SWCNTs for NO detection [4,32,33,34,35].

In this study, we fabricated gold nanoparticles (Au NPs) decorated- SWCNTs-COOH sensing materials and studied their sensitivity to NO in various humidity backgrounds. Acid treatment is a common approach to add carboxylic group to CNTs. This step will also introduce defects in the carbon nanotube network in a controlled manner and facilitate target gas molecules adsorption and charge transfer at the defect sites [36]. CNTs decorated with Au NPs have been extensively studied for gas sensing and researchers have reported enhanced sensitivity toward gaseous species such as H_2_, H_2_S, NO_2_, NH_3_, CO, CO_2_, and ethanol compared to pure nanotube gas sensors [28,30,37,38,39,40,41,42,43,44].

Our work shows a simple drop casting method that introduce a composite of Au NPs and SWCNTs-COOH onto an interdigitated electrode to make a reliable, sensitive, and cost-effective gas sensor with enhanced sensitivity to NO gas at room temperature. The sensor can reliably detect 10 ppb NO in situ in a wide humidity range of 0–97%, which is suitable for dissolved NO detection in the headspace of Sea water using a semipermeable membrane to allow only gas sample (no water) passing through it to a chamber at different undersea levels for the oceanographic study and for human breath analysis in medical diagnosis.

## 2. Materials and Methods

Single-walled carbon nanotubes (>90% purity) were purchased from US Research Nanomaterials, Inc. (Houston, TX, USA). These purified nanotubes were further treated with sulfuric acid (98% wt., Sigma Aldrich) and nitric acid (68% wt., Sigma Aldrich) as described in the literatures to add various oxygen containing groups and opening up of the nanotubes’ caps [45,46,47]. About 30 mg of pristine SWCNTS were taken in a flask and then slowly 40 mL of mixed acid consisting of sulfuric acid and nitric acid with the volume ration of 3:1 was added. The mixture was refluxed at 120 °C for 4 h. The solution was then diluted with de-ionized water and centrifuged to recover the SWCNTs-COOH. The material was further washed with de-ionized water multiple times to bring the pH value of the filtrate to neutral. SWCNTs-COOH were dispersed (0.03% by weight) in DI water. The suspension was sonicated for about 30 min.

Gold nanoparticles were synthesized using the classical, well described Turkevich method [48,49,50,51]. First, all the glassware and magnetic stirrer were thoroughly cleaned with aqua regia (mixture of nitric acid and hydrochloric acid, in a molar ratio of 1:3) and then rinsed with de-ionized water. This step avoids aggregation of residual gold particles during the synthesis procedures [52]. Gold (III) chloride hydrate (HAuCl_4_) and Trisodium citrate (Na_3_C_6_H_5_O_7_) were purchased from Sigma Aldrich (Burlington, MA, USA). HAuCl_4_ solution (1 mM, 80 mL) was taken into a flask and heated to a boiling temperature with a uniform stirring. After reflux started, Na_3_C_6_H_5_O_7_ (38.8 mM, 9 mL) was slowly added. The color of the solution changed from yellow to dark purple. After about 40 min of reflux, the mixture was slowly cooled down to room temperature. Finally, the product was centrifuged to obtain Au NPs and stored in the dark place to minimize the photo induced oxidation.

The hybrid composites as the sensing material were prepared by mixing SWCNTs-COOH with Au NPs (pH 7). First, to optimize the Au NPs loading onto the SWCNTs-COOH network, three compositions were prepared by varying the amount of Au NPs 1%, 5%, and 10% (by weight) in the SWCNTs-COOH dispersion. The composite materials were stirred overnight at room temperature. 

The substrate of the sensor chip was made by grade FR-4 PCB. This sensor chip contains an array of 16 gold printed interdigitated electrode (IDE). The IDEs were microfabricated using screen printing technique on a 2 × 1 cm^2^ chip area and each IDE had a finger width of 70 µm and gap size of 102 µm. Each IDE was manually drop casted with 0.3μL of composite materials. The sensor chip was air dried overnight and with the evaporation of the solvent, the deposited nanomaterials form a network of the composites that bridge the fingers of the IDEs. This nanoarchitecture provides a high surface area and continuous electrical connectivity between the fingers of IDEs. Base resistance of the sensors varied according to Au NPs loading amount. As expected, the conductivity of the sensors increased as amount of Au NPs increased. The base resistance of the sensors varied from ≈640 Ω (10% Au NPs) to ≈15 kΩ (1% Au NPs).

The gas sensing experiments were carried out by sequential exposure of the sensors to various concentrations of certified NO gas in a cylinder (2.5 ppm balanced in nitrogen, Praxair) premixed with either zero air or nitrogen (Praxair). An Environics 2000 (Environics Inc., Tolland, CT, USA) gas blending and dilution system was used for producing desired concentrations of NO at different humidity levels. For the electrical resistance measurement of each sensor channel, the sensor chip was connected to a Keithley 2700 (Keithley Instruments, Inc., Scottsdale, AZ, USA) via an interface board. A constant 400 CCM sample flow of desired concentration of NO gas were introduced to the sensor chip in a small chamber with a Teflon cover which sits on top of the sensor chip to evenly disburse the gas steam to all sensor channels. The experimental setup of sensor testing is discussed in our earlier publication [53]. To monitor temperature and humidity around the sensor area, a surface mount humidity and temperature sensor (Texas Instruments, HDC 1000YPAT) was placed next to the sensor chip under the Teflon cover and humidity was adjusted from 0–97%. All NO gas exposures were introduced after 10 min of nitrogen flow for baseline recording to allow humidity and baseline stabilization. Each NO measurement was consisted of 1 min (0.02–1.5 ppm) exposure followed by a 5 min of nitrogen purge, alternate sample exposure and purge cycles were introduced at room temperature.

## 3. Results and Discussion

### 3.1. Sensor Characterization

**Figure 1:**
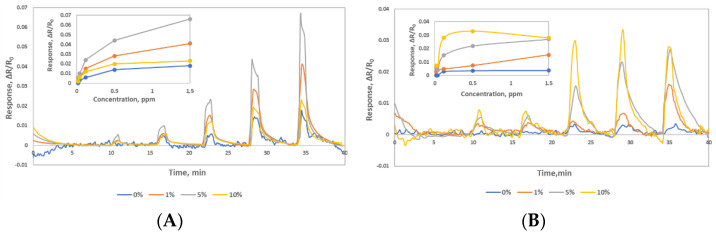
Response of a sensor chip to 0.02, 0.04, 0.12, 0.5 and 1.5 ppm NO gas. Each line represents the composite material made with varying loading of Au NPs onto SWCNTs-COOH. (**A**) sensor response in dry condition (**B**) sensorresponse in 92% RH.

A sensor chip with different composites varying Au NPs loading was prepared. As expected, the conductance of the sensors varied based on the Au NPs loading in the CNTs composites, higher loading increased the conductance [54]. As prepared SWCNTs-COOH (0% Au NP) measured base resistance 30 KΩ, adding 1% Au NPs measured 11KΩ, adding 5% Au NPs measured 1.8 KΩ, and adding 10% Au NPs measured 624 Ω. To find the optimum gold particle to CNTs ratio, sensors were tested with various concentrations of NO gas (0.02–1.5 ppm) in Nitrogen background as shown in Figure 1. All four materials showed sensitive response to NO gas and the 5% AuNPs loaded material showed the highest response in the dry condition, see Figure 1A. This material also showed the highest concentration dependent sensor response in the humid condition, see Figure 1B. SWCNTs-COOH (0% Au NP) only responded to high concentrations of NO gas (0.2–1.5 ppm). The 10% Au NPs composite showed higher response in the humid condition, but the response was not concentration dependent and saturated after 0.12ppm. Therefore, 5% Au NP with SWCNTs-COOH was selected as Material 1 for further study. In humid conditions, Material 1 as prepared in water with pH 7 showed lower response due to the NO gas reacts with water and produce nitrous acid. To take advantage of the acid-base chemistry, we modified the Material 1 by adjusting the composite solution’s pH to 10 to make a basic material—Material 2. Materials with pH > 10 resulted in lower baseline stability. Therefore, we selected Materials 2 prepared in pH 10 solution for our humid study.

**Figure 2:**
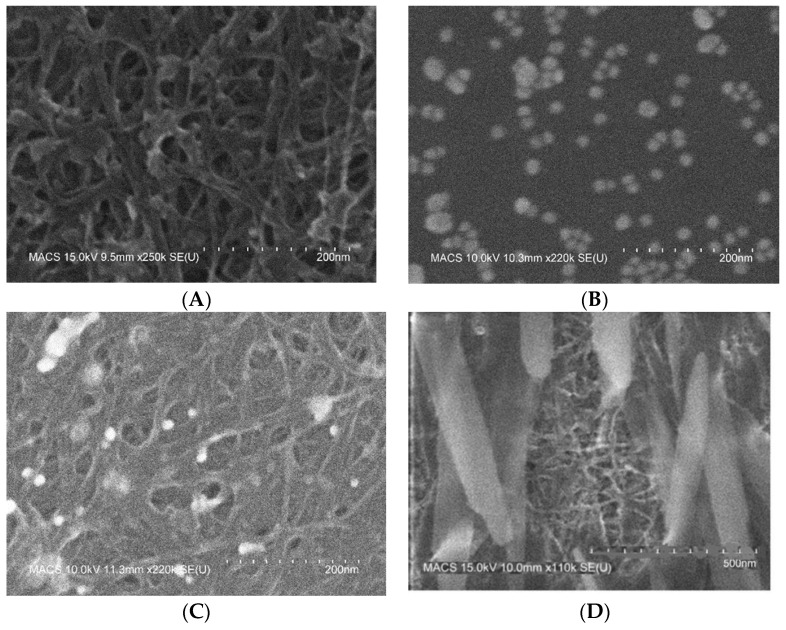
FE-SEM images for materials deposited onto a silicon substrate (**A**) SWCNTs-COOH (**B**) Au NPs (**C**) Composite Material 1 (**D**) composite Material 2.

The images of the morphology of the SWCNTs-COOH, Au NPs, composite Material 1, and composite Material 2 were obtained using Field emission scanning electron microscopy (FESEM) Hitachi S-4800 SEM. The sensing material were deposited on a silicon substrate instead of imaging the chip directly which was made on PCB substrate. As shown in Figure 2A, the acid treated SWCNTs appeared as a tangled network of bundles of multiple nanotubes, which were densely aggregated to make a cluster. The average diameter of the SWCNTs-COOH and composite materials nanotubes appears to be 6-10 nm and the length of the bundles is 0.1–1 μm. The surface was rough, and several fragmentations were observed. This is expected as previous researchers have reported that the strong oxidizing agents, e.g., sulfuric acid and nitric acid etched the graphitic structure and caused the change in the structural integrity [47,55]. The image/morphology of Au NPs in Figure 2B showed the development of spherical nanoparticles with diameter about 15–20 nm. Particles seems to be mostly free of agglomeration. This is an expected size as reported by the previous researchers [48,49,50,51,56]. Turkevich method is well studied by many researchers using the variations in HAuCl_4_/sodium citrate ratio, pH of the solution, and temperature. These factors influence the nanoparticle size and stabilization. Figure 2C showed presence of Au NPs on SWCNTs-COOH. Au NPs can be observed as bright sphere attached to nanotubes. Figure 2D shows presence of NaOH crystals on SWCNTs-COOH-Au NPs network.

**Figure 3:**
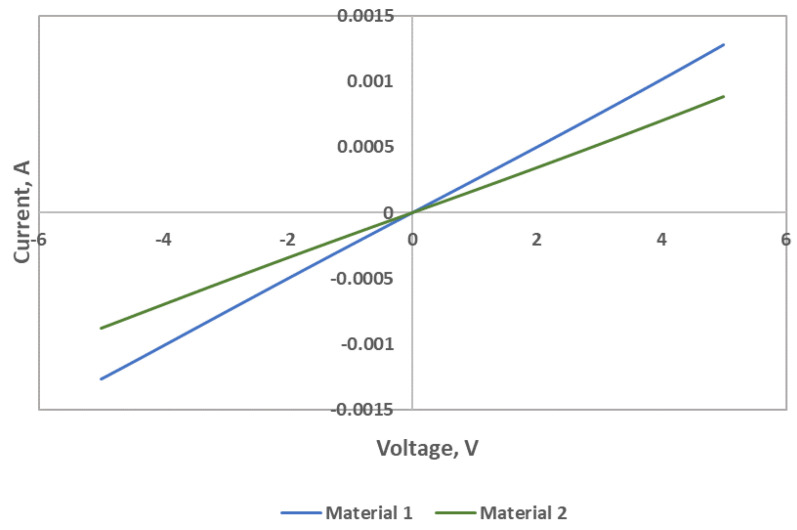
I-V characteristics of the sensing materials: Material 1 and Material 2.

Figure 3 shows I-V characteristics of Material 1 and Material 2. I-V plots were measured using HP Semiconductor Analyzer 4155A. The current was measured when a bias voltage of −5 to 5 V was applied in air environment at ambient room temperature and humidity. Material 1 showed higher conductivity than Material 2.

**Figure 4:**
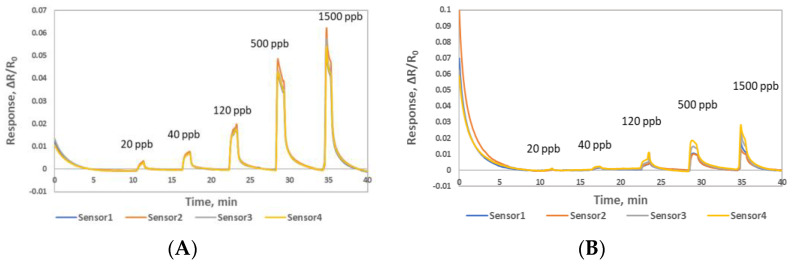

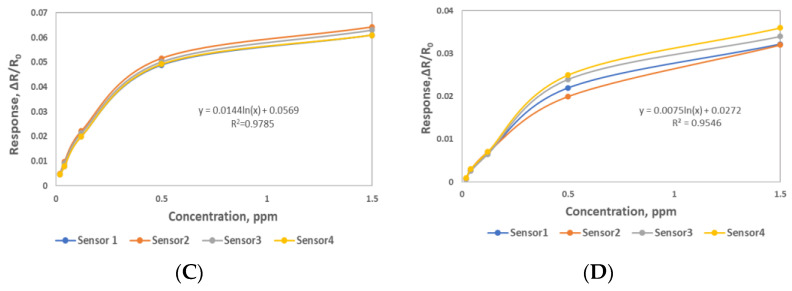
Sensor responses (ΔR/R_0_) to 0.02, 0.04, 0.12, 0.5, 1.5 ppm NO gas at 0% RH (**A**) Material 1 sensors (**B**) Material 2 sensors (**C**) calibration curves of four sensors made from Material 1 (**D**) calibration curves of four sensors made from Material 2.

Typical sensor responses of Material 1 and Material 2 to NO gas at room temperature were obtained, see Figure 4. The four sensors of each material were exposed to NO concentrations of 0.02, 0.04, 0.12, 0.5 and 1.5 ppm at 0% RH and room temperature (≈26 ˚C) at the shown interval. The sensors were purged with a nitrogen at the flow rate of 400 CCM for the first 10 min to obtain a stable baseline and then 5 min purge after each 1 min NO exposures. In the figure, four sensor channels with the identical sensing material are shown as the colored response curves. The response curves plotted here are normalized resistance (R − R_0_)/R_0_, where R_0_ is the baseline resistance right before the NO gas exposure and R is the resistance at any time t during the NO gas exposure. In Figure 4A, the sensors fabricated from the Material 1 showed clear sensitivity to 0.02 and 0.04 ppm NO concentrations, while Material 2 barely showed sensitivity to these lower concentrations with the heavy drifted baseline, see Figure 4B. In addition, Material 2 showed significant baseline drift at the beginning of the measurement. This initial drift is caused by the current applied to the sensors for resistance measurement as well as the shifting from ambient humidity to the cylinder humidity (0% RH). Both sensing materials showed high reproducibility when exposed to different concentrations of the NO gas. The sensor responses of four replicates of Material 1 and Material 2 to various concentrations of NO with the corresponding calibration curves are shown in Figure 4C and Figure 4D, respectively. The sensor response and the concentration relationship were logarithmic, y = a + bLn(C) where y is the normalized sensor response and C is the concentration of NO gas. Sensors response time were <10 s and sensors recovery time were around 1 min. The sensor response variation with four identical sensors to NO gas was calculated by the standard deviation as 1.81% for Material 1 and 5.52% for Material 2. Sensors made from both materials showed good and acceptable reproducibility.

Both composite materials showed sensitivity to NO gas. All materials showed increase in the electrical resistance when exposed to various concentration of NO. Unpaired electron on the NO molecule makes it very reactive and donated it to the CNTs. Because of a lone pair of electrons that can be transferred from NO to carbon nanotubes while the NO adsorbed on Au NPs/SWCNTs-COOH composite, the electron-donation from the target gas leads to a reduction in the number of hole carriers in SWCNTs, shifting of the Fermi level from valence band increasing their separation, and thus increasing the electrical resistance. Both composite materials responded to NO similarly indicating these materials are p-type materials that has been studied by other groups [40,57]. Additionally, for the polar and highly reactive NO gas, the charge transfer mechanism for the sensor response is due to the adsorption of the NO gas molecules onto the SWCNTs bundles where the Au NPs assisted the adsorption, which is mainly attributed by the intertube electron modulation effect because the gas molecule adsorption is stronger on the functionalized surface of the nanotubes than on an interstitial space between the nanotube bundle [58].

**Figure 5:**
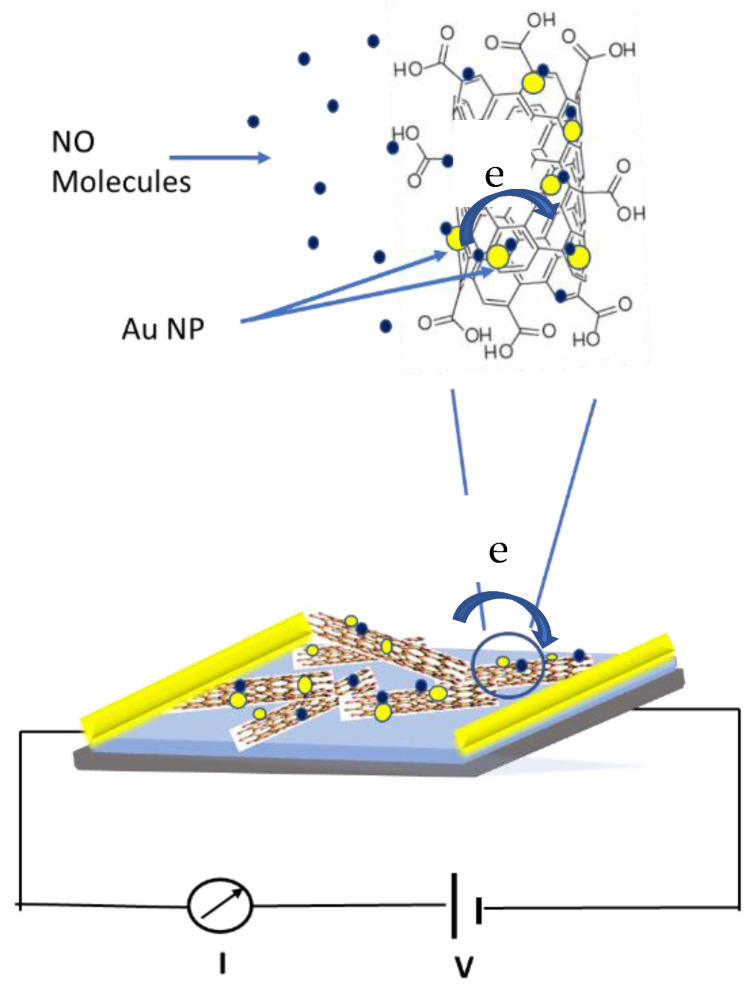
A schematic of possible sensing mechanism.

Gold nanoparticle decorated SWCNTs-COOH showed higher sensitivity to NO gas compared to bare SWCNTs-COOH. The gold nanoparticles acted as the binding sites for NO gas adsorption. The improved response with Material 1 sensors might be attributed to the functionalization of carboxylic groups on the CNTs. Acid treatment introduces COOH and OH groups as well as defect sites on the nanotubes surface [59,60]. Gold nanoparticles will attach to this high energy defect sites compared to clean sp^2^ bonded lattice to lower the energy barrier allowing easier electronic interaction between gold and carbon nanotubes [61]. As shown in Figure 5, when sensor is exposed to NO gas, gas molecules can be adsorbed on the surface of Au NPs, which can lead to decrease the work function of Au NPs. These lower work function sites can enhance the electron transfer from the Au NPs to SWCNTs network, which in further traps the p-type carriers in the CNT network and resulting in the increase in the resistance of the sensor [23,62,63]. However, Material 2 had pH 10 during the functionalization process and that would have deprotonated the -COOH group on the nanotubes and result in the reduced adsorption and hence less interaction with NO gas. Deprotonation would cause electronic structural changes in nanotubes and shifts Fermi level of the nanotubes and as a result the electrical resistance increase is not as much as Material 1 [64].

### 3.2. Humidity Effect on Sensor Response

**Figure 6:**
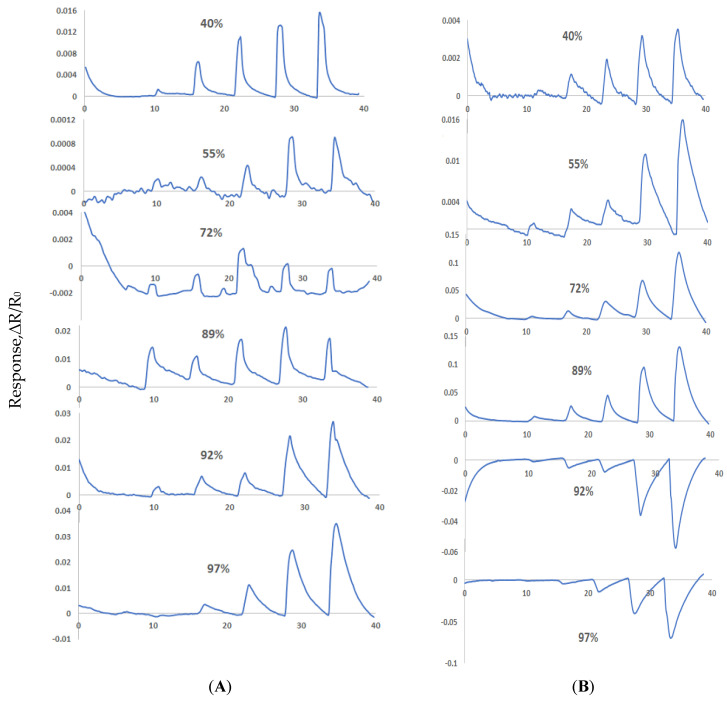

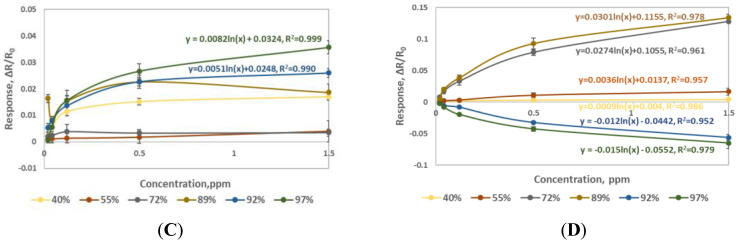
Responses (ΔR/R_0_) to 0.02, 0.04, 0.12, 0.5, 1.5 ppm NO gas in 40%, 55%, 72%, 89%, 92% and 97% RH (**A**) Material 1 nanocomposite sensor response (**B**) Material 2 nanocomposite sensor response (**C**) calibration plots for Material 1 (**D**) calibration plots for Material 2.

Sensors made from Material 1 and Material 2 were exposed to various concertation of NO gas in different relative humidity (RH) background. Relative humidity was set at desired level using Environics gas dilution system and when a stable humidity level was achieved, NO exposures were introduced to the sensors at the time intervals shown in Figure 6. Figure 6 shows that sensor channels with Material 1 and the sensor channels with Material 2 provided varied responses to NO gas at RH of 40%, 55%, 72%, 89%, 92% and 97%. Material 1 showed good sensitivity to NO at 40%, 92% and 97% RH where sensor responses varied according to concentration of NO gas, but in the range of 55–89% RH, Material 1 showed noisy and random responses which was not concentration dependent. Material 2 showed sensitivity to NO gas over the whole relative humidity range of 40–97%. At 40% and 55% RH both material’s responses reduced significantly compared to the results in dry air. This lower sensitivity can be attributed to water molecules interference with the charge transfer process at the functional groups of the materials. Material 1 showed significantly increase in sensitivity to NO gas at RH ≥ 92% and Material 2 at RH ≥ 72%. This behavior of Material 1 and Material 2 in humid environment can be attributed to an additional mechanism occurred that is associated with NO gas dissolution into the water molecules in the sample stream by the following reaction.

NO (g) ⇌ NO (aq)(1)

4NO (aq) + H_2_O ⇌ N_2_O (aq) + 2HNO_2_ (aq) (2)

HNO_2_ + H_2_O ⇌ H^+^ (aq) + NO_2_^−^ (aq) (3)

When NO molecule interacts with water molecule it forms nitrous acid (HNO_2_) which further dissociates into hydrogen ion (H^+^) and nitrite ion (NO^2−^) thus influence the sensing material’s pH. It is reported that NO solubility in neutral media is very low but in alkaline media solubility of NO gas is much higher [65]. Higher NO concentration will result into higher H^+^ ions and thus bring larger change to sensing material’s pH. Material 2 with added NaOH is more basic which will allow higher solubility of NO. As the RH goes higher, dissociation of HNO_2_ is higher due to its alkaline nature which pulls the third equilibrium to the right side and allows more NO molecules to dissolve and thus results in the higher pH change and subsequently higher resistance and therefore higher sensitivity. At higher humidity of 92 and 97%, the sensor response peak direction changed from positive to negative for Material 2. This behavior may be attributed to the combination of limited COOH groups presence and the higher dissociation of HNO_2_ at higher humidity. Higher protonation rate occurring at higher humidly can result into access of H^+^ ion flow beyond the fully protonation of the limited COOH groups present on the nanotubes and that can shift the Fermi level towards the intrinsic Fermi level (n-type) and thus flip the sensor response in opposite direction [66]. This interesting phenomenon needs further investigation.

For Material 1, as it is a neutral material and the NO solubility in neutral media is much lower compared to Material 2. Lower dissociation of HNO_2_ would cause a lower amount of protonation of the -COOH group and thus smaller pH change and subsequently resulted in less resistance change and therefore lower sensitivity at a higher humidity level.

For the practical applications, a humidity sensor needs to be placed with these NO sensors to accurately measure the RH level that will lead to the calibration curves for NO concentration prediction accordingly. Material 1 should be used for NO detection in dry condition and Material 2 should be used for humid conditions (40–89% RH) as it provides a reliable result. For applications where humidity is constant, both sensors can detect ppb level NO concentrations in 90–97% RH.

### 3.3. Air and Nitrogen Environment Effect

**Figure 7:**
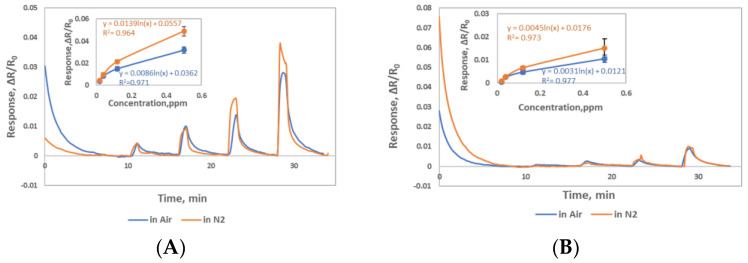
Responses (ΔR/R_0_) to 0.02, 0.04, 0.12, 0.5ppm NO gas (**A**) Material 1 sensors in air and nitrogen (**B**) Material 2 sensors in air and nitrogen.

Sensing materials response to NO gas in air environment was briefly studied. Gas vendor provides NO cylinders always balanced with nitrogen due to reactivity of NO in oxygen environment. In presence of oxygen, NO converts to NO_2_ gas. For this reason, all our experiments were caried out in nitrogen environment. However, we wanted to check our sensor response to NO in air background. We only tested lower concentrations of NO in air. Both Materials were tested for NO concentrations 0.02, 0.04, 0.12 and 0.5 in air and nitrogen in dry environment as shown in Figure 7A,B. Material 1 showed slightly higher sensitivity in nitrogen at higher concentrations but Material 2 showed about similar sensitivity in air and nitrogen. These results are in accordance with the previous reports [15]. In air, oxygen doping will cause Fermi level shift as well as NO will interact with oxygen molecule to make different oxygen containing compounds (NO_2_) and this will result into a different sensing mechanism involving oxygen.

### 3.4. Selectivity and NO Detection Limit

**Figure 8:**
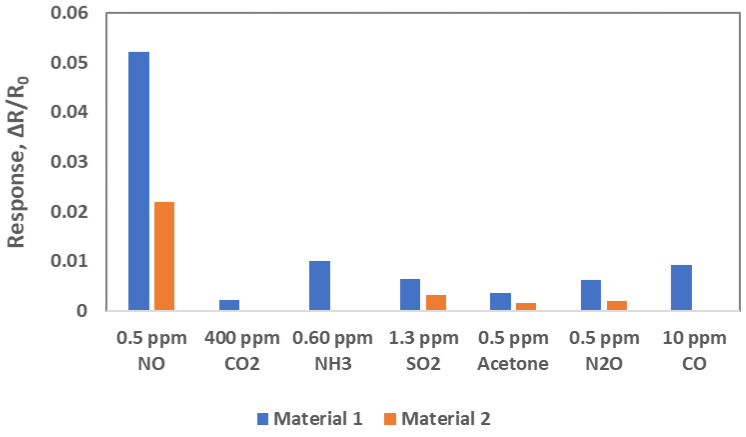
Comparison of sensitivity for Material 1 and Material 2 sensors to commonly interfering concentration of CO_2_, NH_3_, SO_2_, acetone, N_2_O and CO gases at room temperature in air background.

Selectivity of both Material 1 and Material 2 was studied by exposing sensors to CO_2_, NH_3_, SO_2_, acetone, N_2_O and CO gases in air in dry environment. Instead of exposing the sensors to one specific concentration of these gases, we exposed them to the concentrations which are commonly found in the environment. Figure 8 shows normalized sensor responses of both Material 1 and Material 2 to various gases. Material 1 showed much higher response to NO than to other gases. Material 2 also showed higher response to NO and a little response to SO_2_, acetone and N_2_O, and no response to CO_2_, NH_3_ and CO at all. Since the responses of both materials were much higher to NO gas than to the other gases available in their commonly observed concentrations, which indicated these two materials are selective to NO gas. Similar study will be conducted in the humid environment.

**Figure 9:**
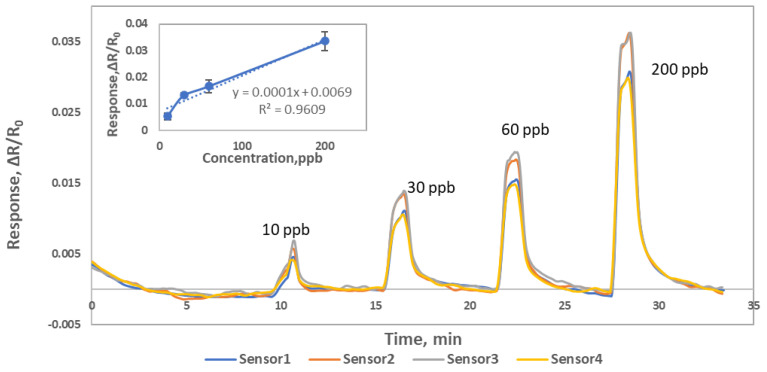
Material 1 response (ΔR/R_0_) to 0.01, 0.03, 0.06, 0.2 ppm NO gas at 0% RH.

To explore detection limit of Material 1, four Material 1 sensors were exposed to 10, 30, 60 and 200 ppb NO concentrations at 0% RH and 600 CCM flow. Material 1 showed clear response to 10 ppb NO concentration, see Figure 9. We see an obvious sensor response to 10ppb and expected or can be extrapolated to lower ppb detection level in N_2_ background.

## 4. Conclusions

We have demonstrated for the first time the use of hybrid Au NPs and SWCNTs-COOH composites as highly sensitive nanomaterials for the detection of NO gas. Our sensing materials can detect 10 ppb NO gas at room temperature in real time and possibly even single digit ppb detection level. Our sensor fabrication technique is simple and cost-effective compared to other methods which involve sputtering, electron beam evaporation, multi-layer deposition, chemical vapor deposition or chemical attachment of preformed metal clusters. Both composite materials showed good reproducibility with 1.81% (Material 1) and 5.52% (Material 2) variation from sensor to sensor. Both composite materials showed sensitive response in high humidity background. A sensor chip containing both Materials 1 and Material 2 composites can detect 10 ppb NO gas in the range of 0–97% RH. Sensors made by both composite materials worked successfully in either air or nitrogen background. Results of selectivity study to different potential interfering gases such as CO_2_, NH_3_, SO_2_, acetone, N_2_O and CO gases indicate the sensors are selective to NO gas. This study demonstrates that tuning the nanoarchitecture and pH value of the sensor materials are the key for improving sensor performance. Our sensors exhibit low ppb level detection, room temperature operation, reproducibility, selectivity, miniaturization, and real time operation and thus meets the needs for the applications in conjunction with a smart phone and other hand-held devices for the application of NO mapping under ocean, breath diagnosis, and in ambient air for environmental monitoring and industrial process monitoring.

## Data Availability

Not applicable.
